# Protein Localization at Mitochondria-ER Contact Sites in Basal and Stress Conditions

**DOI:** 10.3389/fcell.2017.00107

**Published:** 2017-12-12

**Authors:** Nicolò Ilacqua, Miguel Sánchez-Álvarez, Magdalena Bachmann, Veronica Costiniti, Miguel A. Del Pozo, Marta Giacomello

**Affiliations:** ^1^Department of Biology, University of Padova, Padova, Italy; ^2^Mechanoadaptation and Caveolae Biology Lab, Cell and Developmental Biology Area, Centro Nacional de Investigaciones Cardiovasculares Carlos III, Madrid, Spain

**Keywords:** mitochondria-ER contact sites, protein targeting, post-translational modifications, lipid rafts, ER stress

## Abstract

Mitochondria-endoplasmic reticulum (ER) contacts (MERCs) are sites at which the outer mitochondria membrane and the Endoplasmic Reticulum surface run in parallel at a constant distance. The juxtaposition between these organelles determines several intracellular processes such as to name a few, Ca^2+^ and lipid homeostasis or autophagy. These specific tasks can be exploited thanks to the enrichment (or re-localization) of dedicated proteins at these interfaces. Recent proteomic studies highlight the tissue specific composition of MERCs, but the overall mechanisms that control MERCs plasticity remains unclear. Understanding how proteins are targeted at these sites seems pivotal to clarify such contextual function of MERCs. This review aims to summarize the current knowledge on protein localization at MERCs and the possible contribution of the mislocalization of MERCs components to human disorders.

## Introduction

The term “synapse” refers to a site at which two neurons are close enough to communicate to each other. Here, electrical or chemical signals are integrated to determine specific responses, such as the generation of action potentials. The concept of synapse and synaptic integration can be extended to other cells. For example, immunological synapses have been defined for T cells (Norcross, 1984; Paul and Seder, 1994; Grakoui et al., 1999; Bromley et al., 2001; Viola et al., 2010): these are the sites at which signaling cascades originating from T cell Receptors are ultimately “decoded and integrated” to achieve either activation or tolerance.

This concept can be further extended to contact sites between intracellular compartments such as those amongst mitochondria and Endoplasmic Reticulum (ER). Mitochondria-ER contacts (MERCs) are sites in which the surfaces of the two organelles juxtapose at a constant distance, for several nm in length. These contacts can be isolated through subcellular fractionation procedures and the membrane fraction corresponding to the MERCs is known as MAMs (mitochondria associated membranes; Vance, 1990; Rusiñol et al., 1994). Thus, MAMs are the biochemical counterpart of MERCs (Giacomello and Pellegrini, 2016).

Cues comprising information from cell growth signaling, metabolic, and stress-responsive programmes are integrated at MERCs, determining cell wellbeing/homeostasis. Therefore, it is not surprising that the disruption of MERCs has been associated with an ever-growing number of pathologies, as an element contributing to the propagation of functional imbalances across cellular systems—such as lipid imbalance and insulin resistant states (Arruda et al., 2014).

MERCs-associated functions, composition, and extension seem to be tailored to specialized tissues- further stressing their relevance for the fine tuning and integration of multiple functions. But how are MERCs defined and how is their specific composition dictated? Viola et al. (2010) proposed that integration of different signaling steps may promote the rearrangement of lipids within membranes, thus providing specialized platforms at which signals can be generated, amplified, or even blunted. Whether this principle applies to MERCs is a standing question to explore. The latter have been already shown to have “lipid raft”-like properties (Hayashi and Fujimoto, 2010; Area-Gomez et al., 2012), although this aspect needs further clarification. Besides “raft”-like domains, MERCs are characterized by the presence of proteins that either tether the two organelles together or dictate their biological function. These “molecular bridges” appear as electron dense rods in EM images (Csordás et al., 2006). Among the potential tethers identified to date, the most studied in higher eukaryotes is Mitofusin2 (Mfn2), first discovered as a key factor for mitochondrial fusion (Chen et al., 2003). Its presence at the surface of the ER and the evidence that ER-located Mfn2 binds to the OMM located Mfn2 and Mfn1 (de Brito and Scorrano, 2008; Naon et al., 2016) strongly suggested its involvement in the control of MERCs. Another key protein required for MERCs assembly and activity is PhosphoAcidic Cluster Sorting protein 2 (PACS2; Simmen et al., 2005): its ablation decreases the interaction between the two organelles and the activities of the MAMs resident proteins phosphatidyl serine synthase 1 (PSS1) and long-chain fatty acid acetyl-CoA synthase (ACSL4; Piccini et al., 1998; Simmen et al., 2005). A third tethering complex proposed for higher eukaryotes includes the integral ER protein Vesicle-Associated membrane Protein associated protein B (VAPB) and the OMM Protein Tyrosine Phosphatase Interacting Protein 51 (PTPIP51). Interestingly, the VAPB-PTPIP51 tethering complex negatively controls autophagy and is dysregulated in frontotemporal dementia (De Vos et al., 2012; Stoica et al., 2014; Gomez-Suaga et al., 2017).

Although these (and others, for a detailed list please refer to Table 1, and to: Area-Gomez et al., 2012; De Mario et al., 2016) structural components of MERCs have been uncovered, it is nowadays clear that MERCs display cell-specific tissue composition, as highlighted by a number of proteomic analyses (Poston et al., 2011; Horner et al., 2015; Liu et al., 2015; Sala-Vila et al., 2016). While the interest on the biology of MERCs has recently soared, further systematic studies are required to get a complete view of the MERCs toolkit.

**Table 1:** Overview of the main MERCs functions and actors.

**MERCs Function**	**MERCs main players (gene symbol)**	**References**
Ca^2+^ homeostasis	ATP2A1	Chami et al., 2008
	HSPA5	Hayashi and Su, 2007
	HSPA9	Szabadkai et al., 2006
	ITPR	Szabadkai et al., 2006
	MFN2	de Brito and Scorrano, 2008
	PSEN2	Zampese et al., 2011
	PTPIP51	Stoica et al., 2014
	SYGMAR1	Hayashi and Su, 2007
	VDAC	Szabadkai et al., 2006
Lipid homeostasis	ACAT1	Rusiñol et al., 1994
	CAV1	Sala-Vila et al., 2016
	ERLIN 2	Browman et al., 2006
	FACL4	Lewin et al., 2001
	OSBPL	Galmes et al., 2016
	PEMT	Cui et al., 1993
	PTDSS1-2	Stone and Vance, 2000
	REEP1	Cajigas et al., 2012
	SERAC1	Wortmann et al., 2012
	STX17	Hamasaki et al., 2013
	SYGMAR1	Hayashi and Su, 2007
	VAPB	Stoica et al., 2014
Mitochondrial dynamics	DNM1L	Friedman et al., 2011
	FIS1	Iwasawa et al., 2011
	FUNDC1	Wu et al., 2016
	MARCH5	Sugiura et al., 2013
	MFF	Elgass et al., 2015
	MFN2	de Brito and Scorrano, 2008
	MIEF1	Elgass et al., 2015
	MIEF2	Elgass et al., 2015
	PACS2	Simmen et al., 2005
Autophagy/mitophagy	AKT	Betz et al., 2013
	ATG5	Hamasaki et al., 2013
	ATG14L	Hamasaki et al., 2013
	FUNDC1	Wu et al., 2016
	MTOR	Betz et al., 2013
	PARK2	Calì et al., 2013
	PINK1	Cajigas et al., 2012
	STX17 ZFYVE1	Hamasaki et al., 2013 Hamasaki et al., 2013
Immune response	NLRP3	Zhou et al., 2011
	p66Shc	Lebiedzinska et al., 2009
	PML	Giorgi et al., 2010
	PTEN	Bononi et al., 2013
	PTPIP5	Stoica et al., 2014
	PYCARD	Zhou et al., 2011
	RAB32	Bui et al., 2010
	TXNIP	Zhou et al., 2011
ER homeostasis	ERN1	Mori et al., 2013
	SIGMAR1	Hayashi and Su, 2007
	EIF2AK3	Verfaillie et al., 2012
	CANX	Myhill et al., 2008
	ERO1A	Gilady et al., 2010

Notably, while proteins involved in the maintenance of lipid and Ca^2+^ homeostasis can be retrieved at MERCs in basal conditions, some proteins enrich in these sub-compartments only upon stimulation. Thus, MERCs, similarly to membrane rafts, function as platforms for composite signal transduction complexes. How proteins are recruited to these “biological interfaces” and retained there still needs to be clarified and is fundamental to understand MERCs physiological role. This review focuses on this aspect, and aims to highlight the principles determining protein enrichment/translocation at MERCs.

## MERCs functions at a glance

As stated above, mitochondria-ER contact sites (MERCs) appear in electron microscopy (EM) as the parallel juxtaposition of the ER surface to the Outer Mitochondrial Membrane (OMM), at a distance ranging from 10 to 80 nm (Giacomello and Pellegrini, 2016). The length and width of the cleft separating both organelles and the protein composition of the communicating membranes are strictly bound to the processes in which MERCs are involved (summarized in Table 1). A number of recent reviews have already summarized in detail the role of MERCs in different subcellular pathways (Rowland and Voeltz, 2012; De Mario et al., 2016; Eisenberg-Bord et al., 2016; Prudent and McBride, 2017). Here, we will just provide a quick overview of the main MERCs functions.

The most established roles of MERCs pertain to their contribution to lipid and Ca^2+^ handling. Almost three decades ago, (Vance, 1990) highlighted the importance of MERCs for lipid homeostasis. Indeed these contact sites shape the specific route for phospholipid interconversion, allowing for the synthesis of phosphatidylethanolamine and phosphatidylcholine from serine and contributing to the composition of mitochondrial membranes (Rusiñol et al., 1994; Vance, 2014). These MERCs-associated routes may turn essential under restrictive conditions such as ethanolamine deficiency (Flis and Daum, 2013). Notably while the synthesis of cholesterol and its precursors, minoritary components of the OMM, is located at the ER, they can be converted into other molecules such as steroid hormones in MAMs (Bosch et al., 2011b; Sala-Vila et al., 2016). Thus, mitochondria-ER contacts appear as the sites at which coordination among lipid homeostasis and other cell functions occurs.

MERCs are also the site of Ca^2+^ exchange between the two organelles: they host a protein complex composed of the inositol triphosphate receptor (IP3R), the voltage-dependent anion channel (VDAC) and the chaperone grp75 (Szabadkai et al., 2006), which allows for rapid mitochondrial Ca^2+^ uptake through the Ca^2+^ Uniporter. The efficient shuttling of Ca^2+^ between both organelles depends on the width of the cleft that separates them: an optimal length of 15–25 nm allows both the assembly of the IP3R-grp75-VDAC complex and a fast Ca^2+^ exchange; on the contrary, a distance below 10 nm impedes the formation of the complex due to steric hindrance (Csordás et al., 2006). On the other hand, a distance above 25 nm would decrease the Ca^2+^ diffusion rate and hence blunt mitochondria Ca^2+^ uptake (Giacomello and Pellegrini, 2016). Mitochondral Ca^2+^ levels impinge on the activity of pyruvate, isocitrate, and α-ketoglutarate dehydrogenases (Denton et al., 1972, 1978), and hence on cell bioenergetics (Cárdenas et al., 2010). On the other hand, if pronounced, a sustained mitochondrial Ca^2+^ uptake can be read as a cell death signal able to trigger permeability of the mitochondrial membranes and opening of the permeability transition pore (PTP) (Bernardi et al., 2001; Hurst et al., 2017). Thus, MERCs also contribute to determine the cell fate (Simmen et al., 2005; Bui et al., 2010; Iwasawa et al., 2011; Prudent and McBride, 2017).

More recently, a bunch of additional functions have been ascribed to MERCs. For example they have been proposed as the site of autophagosome formation, thus playing a key role in autophagy (Hamasaki et al., 2013; Martínez-Pizarro et al., 2016). MERCs appear to couple mtDNA synthesis with mitochondrial division, that is also regulated by the interaction between mitochondria and ER (Friedman et al., 2011; Elgass et al., 2015; Lewis et al., 2016), and can behave as a scaffold that ultimately coordinates immune signaling and inflammasome formation (Lerner et al., 2012; Horner et al., 2015).

Finally, in yeast, mitochondria-ER interaction appears also fundamental for appropriate maintenance of cellular iron homeostasis and mitochondrial biogenesis (Wu et al., 2016; Ellenrieder et al., 2017; Xue et al., 2017).

## Subcellular localization: targeting sequences and more

**Figure 1:**
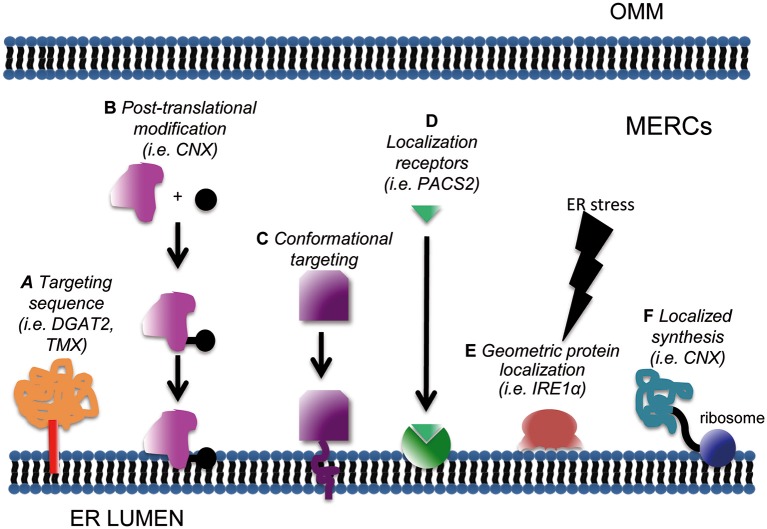
Schematic of subcellular mechanisms for protein targeting at MERCs. Enrichment at mitochondria-ER interface can be achieved through classical targeting sequences **(A)**; post-translational modifications such as phosphorylation, acetylation or sumoylation **(B)**; conformational targeting **(C)**, localization signal receptors **(D)**, geometric protein localization **(E)** and localized synthesis **(F)**.

Protein subcellular distribution relies on several mechanisms (Figure 1). The most common is the presence of a targeting peptide within the protein, which determines its sorting to specific sites. Two questions must be considered in this case: whether specialized or “consensus”-based mechanisms exist, and whether they are subjected to regulation.

So far a robust consensus motif targeting proteins to MERCs has not been defined. Localization at the OMM or ER surface appears sufficient for a protein to be retrieved at MAMs, in a proportion that varies depending on the cell type, culture conditions, oxidation state, or specific metabolic status of the cell. For example, ~90% of the chaperone calnexin (CNX) is homogenously spread in the ER in basal conditions, concentrating at MERCs up to 70% under specific (stress) stimuli (Lynes et al., 2012). To date, only a few putative MERCs-targeting signals have been identified: this is the case for example of a stretch of 67 aa in the cytoplasmic N-terminus of DGAT2 (Stone et al., 2009). Another peculiar motif resides in the transmembrane and cytosolic domains of the transmembrane thioredoxin protein TMX, which are “necessary and sufficient” to ensure TMX accumulation at MAMs (Lynes et al., 2012). As stated above, a unique MERCs-targeting motif has not been identified yet. This could depend on the requirement of post-translational modifications (PTMs) and/or conformational determinants. PTMs as mechanisms for the regulated targeting of proteins at MERCs will be discussed separately in the following section, since a number of evidence substantiating this possibility have been already reported. Another mechanism both hampering the identification of “consensus target motifs” and rendering “alternative compartmentalization” possible might reside in mRNA processing. This remains still a hypothesis, since evidence for the existence of alternative splicing programmes dictating protein localization at MERCs is still lacking.

Another hypothetical MERCs-targeting mechanism would be the existence of “conformational” motifs. That is, a domain composed not by a linear stretch of amino acid, but by a functional interaction surface determined by the rearrangement of the 3D structure of the protein. The possibility of a “conformational” domain appears particularly intriguing for MERCs, since it would provide a mean for switchable recruitment of proteins. If this possibility holds true, it would perfectly match with the highly plastic lipid environment of MERCs. In fact, one of the means for PTMs-regulated targeting to MERCs might rely on such conditional conformational state (see below). Molecular threading for “tridimensional alignment” has been classically very challenging in terms of computational power requirements, but recent advances may ease these approaches to study conformational MERCs-targeting domains.

Another widespread mechanism for regulated protein compartmentalization relies on masking of localization domains: interaction with other partner molecules may confine them into the cytosol or other subdomains. This mechanism usually keeps “silent” (i.e., inactive) a certain protein until its release, which induces its re-localization at sites where specific interacting partners and/or target functions are (Bauer et al., 2015). Theoretically the concept of domain masking could be extended also to MERCs: targeting motifs would be exposed only if the specific function of the protein of interest is needed at these sites.

Subcellular targeting relies also on appropriate “localization signal receptors” (Bauer et al., 2015). This term denotes the presence of a sequestering/scaffolding protein able to bind its ligand and restrict its diffusion. Sequestration of a protein implies that the density of binding sites within a subcellular domain is high enough to significantly limit its mobility toward other locations. Interestingly, the affinity of a localization signal for its receptor can be modulated, especially through PTMs such as phosphorylation, lysine acetylation, or SUMOylation of either the receptor or of the ligand. Changes in the affinity of this interaction can either increase or decrease the compartmentalization of the ligand, by unveiling or, alternatively by masking, any signaling peptide. Thus, PTMs would exert their function not only by modulating the activity of proteins, but also by controlling their localization. As a consequence, they can shape the composition (and hence the function) of subcellular compartments. As to MERCs, an example of localization signal receptor is PACS2, which mediates the localization and enrichment of the CNX at these sites (Myhill et al., 2008). It is interesting to note that not only proteins, but also lipids and phospholipids could behave as localization receptors. For example, it has been shown that phosphatidylinositol (3,5) diphosphate can act as a membrane-targeting molecule, mediating the binding of different proteins to biological membranes (Ferguson et al., 2009; Salminen et al., 2013). Due to the special lipid composition of MERCs, we further elaborate in a separate section on this topic (see below).

Another common protein targeting strategy is “localized synthesis” (Kejiou and Palazzo, 2017). mRNAs encoding for a given polypeptide can be localized to specific subcellular domains where they are either kept silent, waiting for specific stimuli to trigger translation, or efficiently translated if necessary in basal conditions (Kejiou and Palazzo, 2017). Classical examples of spatial protein segregation by localized synthesis can be found in neurons (Rangaraju et al., 2017). Here, localized production is required to quickly shape the response of neurites to the signals coming from synapses. Interestingly, a study conducted to describe the local transcriptome in the synaptic neuropil of CAI Hippocampus (i.e., the mRNA enriched in this specific cell subdomain) highlighted the presence of MERCs resident or regulatory proteins, among which also calnexin, mTOR, Pink1, presenilin2, REEP1, Sigma-1 Receptor, α-synuclein, VAPB (Table S10 of Cajigas et al., 2012). These data support the hypothesis that local translation also contributes to the plasticity of MERCs, that with their activity could in turn match the needs of specialized cell structures.

Finally it's worth mentioning two additional mechanisms, although yet unproven for protein targeting at MERCs. A first one has been termed “geometric protein localization” (Ramamurthi et al., 2009; Updegrove and Ramamurthi, 2017). Mostly studied in bacteria, it relies on the ability of some proteins to “sense” membrane curvature and bind to specific geometric cues. In this case protein localization will be dictated by the shape of the membrane, independently of its composition (Hatzakis et al., 2009; Bhatia et al., 2010). These proteins are often characterized by membrane-binding amphipathic helices (Updegrove and Ramamurthi, 2017) that recognize even small changes in the curvature of membranes and enrich at these special sites. Interestingly, some curvature-sensing proteins have been reported in eukaryotes: this is the case of dynamins (Ramachandran and Schmid, 2008), cytochrome b5 (Taylor and Roseman, 1995), and interestingly, the ER stress-transducer IRE1 (Halbleib et al., 2017; see below). Recently, it was also reported that membrane-anchored proteins can efficiently sense membrane curvature, the latter being an additional mechanism for their efficient clustering (Hatzakis et al., 2009). As to MERCs, so far they have been characterized and described according to specific parameters: the relative length of the ER surface portion that run in parallel to the OMM and the width of the cleft that separates the two organelles (Giacomello and Pellegrini, 2016). Whether they are characterized by a particular membrane curvature range remains, at least to our knowledge, to be defined. The study of “geometric protein localization” at MERCs remains also challenged by the lack of appropriate readouts, compatible with the manipulation of such properties specifically at those sites.

The second mechanism, already shown to operate in the context of ER segregation and partitioning in yeast, pertains to the establishment of “protein boundaries” able to restrict the lateral diffusion of other membrane components (Chao et al., 2014). In this case, cytoskeleton-associated proteins (septins) are the effectors of such compartmentalization. Intriguingly, MERCs constitute in some experimental models points for actin- and microtubule-assisted mitochondrial fission (Ji et al., 2015; Prudent and McBride, 2016). So far, no clear connection has been established between MERC composition and cytoskeletal components, nor septins.

## Post-translational modification: “localization” or “relocation” at MERCs?

PTMs are normally used by the cell to modulate the activity, stability, interaction profile and/or subcellular segregation of proteins. MERCs components are no exception: substantial evidence exists for multiple PTMs fine-tuning their properties, including their localization. As such, most MERCs proteins subjected to PTMs are characterized by (at least) a dual localization: either they have a broad subcellular distribution (for example, at the cytosol, or at the ER, or at the OMM), and then they enrich at MAMs, or the reverse (i.e., they appear located at MERCs, and upon PTMs, they redistribute to other subcellular compartments).

The first possibility applies to CNX. Its binding to PACS2 appears not sufficient for its enrichment at MERCs (Myhill et al., 2008), palmitoylation being the additional trigger necessary for its complete relocation at these interfaces (Lynes and Simmen, 2011). Examples have also been described for the second scenario. Impairment of ER oxidizing conditions causes Ero1α to lose its MAMs localization (Gilady et al., 2010), while heme oxygenase-1 relocates from MAMs to rough ER in the absence of palmitoylation (Lynes and Simmen, 2011). Another example is N-myristoylation: when the carbohydrate-binding protein starch binding domain-containing protein 1 (Stbd1) undergoes this modification, it is mostly retrieved on the ER, its wild type form being on the contrary enriched at MAMs “by default” (Demetriadou et al., 2017).

Generally speaking, PTMs also represent a switch for the activity of several enzymes. Thus, PTMs might control MERCs activity in a dual fashion: on one side, through a modulatory function, by directly enhancing or decreasing the activity of a MERCs-resident enzyme; on the other, by enhancing the contribution of MERCs to a subcellular process through re- or delocalization of specific proteins. Transient PTMs-dependent recruitment at MERCs may explain, at least in part, why changes in the expression levels of some proteins have a profound effect on ER-mitochondria apposition, despite they are not enriched in purified MAM fractions (Wieckowski et al., 2009; Eisenberg-Bord et al., 2016; Naon et al., 2016). Such an example has recently been reported for a novel OMM-ER tethering complex composed by two proteins broadly distributed to the OMM and ER surface: SYNJ2BP and RRBP1 (Hung et al., 2017). These data support the hypothesis of “auxiliary tethers,” according to which some proteins would not be strictly necessary to form a (functional) contact site, but they would be able to do so whenever their MERCs-related function was needed (Eisenberg-Bord et al., 2016).

## “lipid raft”-like behavior as a mechanism for compartmentalization at MERCs

**Figure 2:**
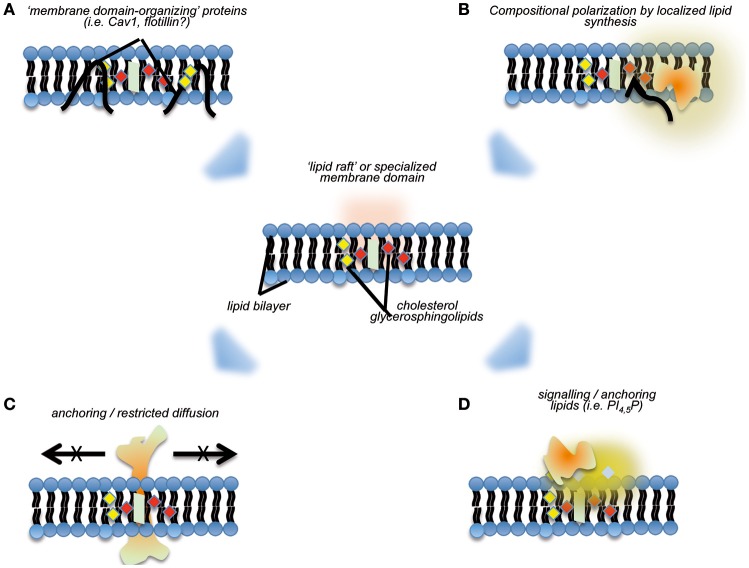
Cartoon summarizing aspects pertaining to the organization of “nanodomains” on membranes, and their potential effects regarding recruitment of specific activities. “Lipid raft”-like domains may be stabilized by specialized membrane-binding proteins, such as caveolins **(A)**, and/or through directional local synthesis and accumulation of specific lipid species **(B)**. The subsequent definition of such membrane nanodomains may preclude lateral diffusion of specific transmembrane proteins **(C)**, and or act as “molecular beacons” for the specific recruitment of proteins **(D)**.

Another layer for regulating MERCs compartmentalization comes from the distinct lipid composition of the membrane domains delimiting MERCs (Figure 2). The concept of dynamic membrane nanodomains or “lipid rafts” initially introduced by Simons and van Meer (1988) almost 30 years ago states that one of the key properties of such membrane patches is the efficient accruing and stabilization of transmembrane proteins and membrane-associated activities (Simons and Sampaio, 2011). Correspondences between mitochondria-ER contacts and generic lipid rafts have been highlighted (Area-Gomez et al., 2012; Annunziata et al., 2013): MAMs have a significantly higher content in cholesterol and sphingolipids as compared with bulk ER membranes (Annunziata et al., 2013; Vance, 2014; Sala-Vila et al., 2016); they seem to have a lower degree on curvature as compared with surrounding ER regions (Rowland and Voeltz, 2012).

The “lipid raft”-like organization of MERCs could help to explain some of their properties and functions. As mentioned above, it would induce the stabilization and limitation of lateral diffusion of ensembles of membrane proteins, or “polarization”—a property that would benefit the initial localization and subsequent sequential assembly of tethering complexes. The full collection of MERCs components in higher eukaryotes remains to be listed, but certain properties of the better-defined tethering complexes in yeast (termed ERMES) support this concept. First, ERMES components exhibit aberrant distribution if expressed in the absence of their partners (Kornmann et al., 2009, 2011; Stroud et al., 2011). Second, analysis of their structure revealed that their membrane-binding domains are fully functional only if they form complexes with appropriate stoichiometry, and only in this case they can recognize organelle contact sites. Thus, it seems that (a) MERCs lipid composition is an essential, but not a sufficient, feature to ensure MERCs targeting; and (b) cooperative recruitment may ensure the regulated, reversible assembly of tethering complexes. The differential lipid composition of MERCs appears critical for the targeting of specific proteins to this subcellular compartment in higher eukaryotes, a major example being constituted by the Prion protein (PrP^C^). While in basal conditions PrP^C^ localizes mostly at the plasma membrane, pro-apoptotic stimuli can induce its relocalization to the MAMs, transducing a pro-death signal from the surface into the cell (Mattei et al., 2011).

Local synthesis and modification of lipids are hallmarks of lipid rafts. These have been proposed as potential mechanisms for the formation and stabilization of these nanodomains: a directional flux of specific lipid building blocks might favor *per se* the establishment of regions characterized by differential composition (Simons and Sampaio, 2011). A major subset of processes enabled by MERCs comprises lipid anabolism and trans-organelle transport of lipids (a classical example being phospholipids, see above). Furthermore, recent surveys highlight the enrichment of lipid metabolism functions at MERCs, including cholesterol synthesis and modification and fatty acid catabolism (Sala-Vila et al., 2016). A still open question is whether such local lipid metabolism sustains the differential composition of MERCs or not. Intriguingly, and further related to these observations, membrane proteins typically ascribed as lipid raft scaffolds/organizers, such as caveolin-1 (Cav1) are specifically enriched at MERCs. Cav1 is a cholesterol-binding protein famous for its role as an essential scaffold of plasma membrane nanodomains named *caveolae* (Parton and del Pozo, 2013). However, Cav1 also assembles in oligomers in the ER and determines cholesterol trafficking across subcellular compartments-including MERCs (reviewed in Bosch et al., 2011a). Cav1 genetic ablation is associated with increased cholesterol content and altered composition of MAMs derived from hepatocytes: Cav1 absence preferentially affects MAMs components involved in cholesterol and fatty acid metabolism, thus stressing the importance of lipid precursor fluxes for the organization and stabilization of these organelle contacts (Sala-Vila et al., 2016).

Altogether, these evidences favor another emerging function of MERCs, acting as “gauges” for lipid homeostasis in the cell. Similarly to plasma membrane lipid rafts, MERCs are sensitive to conditions disrupting or causing imbalances in lipid metabolism and membrane composition (Zhuang et al., 2005; Vance, 2014). Since MERCs are a platform in which many cell pathways converge, their “design” as elements highly sensitive to changes in lipid homeostasis allows for the integration of virtually all those signaling networks with lipid homeostasis. Thus, their dynamics might contribute substantially to phenomena such as dyslipidemia-associated modulation of proteostasis (see below) or insulin resistance (Arruda et al., 2014; Tubbs et al., 2014). Indeed, key regulators of the Pi3K/AKT/mTOR pathway have been reported to specifically localize at MERCs (Betz et al., 2013; Bononi et al., 2013). It is likely that the lipid raft-like properties of MERCs drive such recruitment and that of additional molecular beacons like phosphatidylinositol phosphate species (Hill et al., 2002; Goswami et al., 2005; Simons and Sampaio, 2011).

## UPR and ER stress signaling at MERCs

Besides being the site for the synthesis, folding, and maturation of secreted and organelle-targeted proteins (Braakman and Bulleid, 2011), the ER also allocates other essential tasks, including lipid homeostasis and mobilization, red/ox control and Ca^2+^ flux regulation. Therefore, the ER constitutes a “hub” through which specific imbalances (i.e., dyslipidemia) can be easily propagated to other cellular systems, underpinning complex pathogenic processes such as obesity-related diseases and cancer. Eukaryotes have evolved a complex surveillance system to cope with functional imbalances in the ER, generally termed ER stress: the Unfolded Protein Response (Ellgaard and Helenius, 2003; Chakrabarti et al., 2011; UPR). UPR regulates either pro-survival programmes, aimed at enhancing ER capacity and/or lowering its functional demand; or pro-death pathways, in case of sustained unresolved ER stress (Ron, 2002; Rutkowski and Kaufman, 2004; Naidoo, 2009).

The UPR includes three signaling branches, associated with three ER-resident transmembrane transducers. The first is the Activation Transcription Factor-6 (ATF6). ATF6 is translocated to Golgi membranes, where it is sequentially cleaved by the S1P and S2P proteases (Haze et al., 1999). This yields an N-terminal fragment which acts as a leucine-zipper transcription factor and drives the expression of adaptive programmes. ATF6 signaling leads to the induction of the turnover system “ER Associated Degradation” (ERAD) and of ER chaperones (for a more detailed overview of UPR and ERAD, please refer to Yoshida et al., 2000; Okada et al., 2003; Galehdar et al., 2010; Tsai and Weissmann, 2010; Smith et al., 2011; Hetz, 2012; Arensdorf et al., 2013). The second branch relies on PRKR-like endoplasmic reticulum kinase (PERK), one of the four eIF2alpha-kinases expressed in higher eukaryotes. Upon “sensing” alterations in ER function or integrity through its luminal domain and its transmembrane segment, PERK oligomerizes and becomes catalytically active, repressing mRNA pools and thus reducing the ER load (Harding et al., 1999). PERK activation also favors translation of the activation transcription factor 4 (ATF4), which controls the expression of master regulators of cell survival and apoptosis (Lu et al., 2004). The third and most conserved UPR transducer is the inositol-requiring enzyme 1 (IRE1, of which two isoforms exist, being IRE1α the essential and most ubiquitous one; Tirasophon et al., 1998). IRE1 catalyzes the unconventional splicing of the X-box binding protein 1(XBP1) mRNA, yielding to a potent transcriptional activator that orchestrates adaptive programmes like the physical expansion of the ER itself (Harding et al., 1999; Yoshida et al., 2001; Calfon et al., 2002; Hetz et al., 2006).

As an integral part of ER, it is not surprising that MERCs function and structure are linked to UPR signaling. IRE1 has been found in MAMs, and in turn the ER-mitochondrial interaction significantly impacts on its activation (Mori et al., 2013). In particular, upon acute ER stress, the MERCs-resident chaperone Sigma-1 receptor (SigR1) stabilizes IRE1α, thus favoring its activation and UPR initiation (Hayashi and Su, 2007; Mori et al., 2013). PERK has also been retrieved in MAMs (Verfaillie et al., 2012), where it directly associates with Mfn2. This interaction seems to increase the activation threshold of PERK (Muñoz et al., 2013). Further, conditions disrupting MERCs, such as Mfn2 knockdown, are associated with a sustained activation of some UPR/ER stress response transducers even in basal conditions (Ngoh et al., 2012; Sebastián et al., 2012; Schneeberger et al., 2013). Recent evidence suggests that changes of MERCs dynamics can influence not only UPR triggering thresholds and amplitude, but also UPR shutdown dynamics. For example, the MERCs stabilization appears as an essential component to induce IRE1 shutdown during ER stress recovery (Sanchez-Alvarez et al., 2017). Conversely, MERCs also coordinate cell functioning and UPR activation: increased coupling of ER and mitochondria accompanies early phases of ER stress and sustains the metabolic adaptations necessary for the cell to cope with non-physiological conditions (Bravo et al., 2011). Further potential ties between MERCs and adaptive UPR signaling pertain to activities determining red/ox potential in the ER. For example, protein disulfide isomerases such as PDIA6 accrue at MERCs (Vance and Vance, 2009) and regulate IRE1 activation (Eletto et al., 2014, 2016). ER stress associated with ROS dysregulation is likely transduced by the PERK-dependent branch at MERCs too (Verfaillie et al., 2012). Hence, the confinement of UPR transduction at MERCs appears fundamental for the integration of the UPR response with multiple signaling pathways.

Does ER homeostasis surveillance influence MERCs composition? Specific adaptations at MERCs take place during ER stress. In these circumstances expression levels of CNX at the plasma membrane decrease (Wiest et al., 1995; Okazaki et al., 2000), increasing in parallel at MAMs (Myhill et al., 2008; Lynes et al., 2012). Another prominent example are programmes favoring cell apoptosis in the face of unresolved or excessive ER stress. Sustained PERK activation contributes to stabilize MERCs, enabling lipid peroxidation at the mitochondrial membrane- which in turn enhances expression and/or mitochondrial recruitment of proapoptotic regulators such as Bax and Ca^2+^ uptake (McCullough et al., 2001; Puthalakath et al., 2007; Galehdar et al., 2010; Verfaillie et al., 2012). A peculiar case of MERCs targeting regulated by UPR is embodied by IRE1 and PERK themselves. As stated above, they continuously monitor misfolded protein levels in the ER lumen through specialized domains. Deletion mutants of yeast IRE1 and of vertebrate IRE1 and PERK lacking luminal domains appear insensitive to acute protein misfolding, but retain sensitivity to conditions altering ER composition or physical properties, such as increased global acyl chain saturation or cholesterol content (Brodsky and Skach, 2011; Volmer et al., 2013; Volmer and Ron, 2015). At least in the case of IRE1, such “membrane monitoring” relies on the features of its transmembrane domain (Halbleib et al., 2017; Kono et al., 2017). The latter mechanism is likely to determine the segregation of UPR transducers to MERCs, possibly in combination with other regulatory layers such as transient dimerization or conformational changes. Recruitment of these UPR sensors at MERCs further contributes to the integration between UPR signaling and cell metabolism (Walter and Ron, 2011).

## Disrupted MERCs localization: potential impact in human disorders

In general, it is well-established that defective subcellular localization can either alter the activity of a protein and/or the subcellular processes in which it is involved. If we extend this concept to MERCs and we take into account that they participate in a myriad of essential process (see above), it seems obvious that their alteration or adaptation to stress conditions could both worsen and propagate imbalances across cellular systems—a phenomenon that appears to be common-place for complex diseases. For instance, the disruption of general mechanisms impacting on MERCs, such as protein palmitoylation, could simultaneously affect their integrity and that of other cellular functions. This could be the basis of phenotypic variability and epistatic effects across many different disorders, ranging from schizophrenia and other neurodegenerative disorders to tumor development (Giorgi et al., 2010; Mórotz et al., 2012; Sander et al., 2015).

MERCs dysfunctions could be caused not only by mutations of proteins that exert their function at these sites, but also by impaired targeting of MERCs-resident proteins. The subsequent pathological conditions associated to MERCs defects will be more evident in tissues where that specific MERCs protein is mostly expressed/active. This is the case, for example, of CNX. CNX acts mostly as a chaperone for glycoproteins, which are key molecules for the development and maintenance of myelin structure (Denzel et al., 2002; Quarles, 2002). Hence, it is predictable that defective CNX would cause myelinopathy: this is actually the case, as confirmed in CNX knockout mice (Kraus et al., 2010). Demyelination has diverse causes, such as for example mutations in myelin basic proteins or altered activity of enzymes responsible for the production of cholesteryl esters: defective MERCs activity should be added to the list of possible mechanisms underlying it.

As reported above, MERCs have been implied in metabolic diseases, like obesity and diabetes (Tubbs and Rieusset, 2017). Hepatocytes from obese mice are characterized by increased coupling between mitochondria and ER, and the consequent mitochondrial Ca^2+^ overload is paralleled by higher mitochondrial reactive oxygen species (ROS) production and abnormal glucose metabolism (Arruda et al., 2014). This phenotype can be ameliorated upon silencing of PACS-2 and IP3R1, leading to lower cell stress and increased glucose tolerance (Arruda et al., 2014).

Another MERCs protein, Mfn2, besides being the genetic cause of an inherited peripheral neuropathy (Charcot Marie Tooth 2a), has also been associated with metabolic dysfunctions (Sebastián et al., 2012; Boutant et al., 2017). A recent study highlighted that metabolic transitions in liver are accompanied by changes in the MERCs structure (Sood et al., 2014), further suggesting that MERCs play an active role in metabolic processes: hence, even mild dysfunction of MERCs could exacerbate a given pathological condition.

Downregulation or mutations of a protein that regulates broad physicochemical properties of MERCs may alter the recruitment or stability of defined subsets of MERCs components, thus preferentially impacting specific functions or metabolic routes. An example of such scenario is showcased by models of genetic deficiency in Cav1 protein. Quantitative proteomic profiling of MAM fractions purified from livers of Cav1KO mice shows a depletion of steroid metabolism and fatty acid catabolism regulators (Sala-Vila et al., 2016). It remains to be elucidated whether these changes are due to aberrant membrane composition (i.e., high free cholesterol), and which is their contribution to the metabolic phenotypes associated with Cav1 deficiency (i.e., lipodystrophy and metabolic inflexibility; Bosch et al., 2011b; Fernández-Rojo et al., 2013; Parton and del Pozo, 2013). MERCs-associated lipid metabolism might be of relevance not only for metabolic phenotypes: it could add to the pathogenesis of neurodegenerative disorders like Alzheimer's disease (AD). One of the main gene products associated to familial cases of AD (presenilins, PSs) is enriched in MAMS, although the link between MAMs and sporadic AD is less obvious (Zampese et al., 2011). Importantly, a genetic connection exists between phospholipid/cholesterol dyshomeostasis and AD (Mapstone et al., 2014; Chang et al., 2017). Further, inhibition of cholesterol transport impairs PSs localization at the ER, inducing their accumulation in vesicles and enhancing the production of the main component of AD neurofibrillary tangles, Aβ (Runz et al., 2002). Finally, an apolipoprotein E variant associated with higher risk of lipid metabolism-associated disorders (ApoE4) specifically alters MERCs lipid metabolism and favor AD-like changes *in vitro* (Area-Gomez et al., 2012).

Another interesting example of a potentially pathological mislocalization of a MERCs component is C19orf12. Mutations in this protein, whose physiological function is yet unknown, are the genetic cause of Mitochondrial Membrane Protein Associated Neurodegeneration (MPAN, Hartig et al., 2013). This severe, early-onset pathological condition is characterized by optic atrophy, generalized dystonia, neuropathy, and psychiatric symptoms. Interestingly, C19orf12 has been retrieved at mitochondria, ER and MAMs, and its mutated forms appear to mislocalize. The evidence that fibroblasts from MPAN patients are characterized by higher mitochondria Ca^2+^ uptake suggest that this protein somehow regulates MERCs function, its localization likely causing enhanced ER-mitochondria Ca^2+^ transfer and hence increased sensitivity to apoptosis (Venco et al., 2015). Notably, C19orf12 mutations have been linked to Parkinson's Disease (PD), strengthening the possibility that PD is linked to defective MERCs function. This hypothesis is substantiated by a number of additional findings. For example α-synuclein (α-syn), a protein whose mutations are linked to PD, has been also retrieved in MAMs (Eliezer et al., 2001; Jao et al., 2008; Guardia-Laguarta et al., 2014). The group of E. Schon demonstrated that mutated α-syn has lower affinity for MERCs, thus challenging the theory that mutant α-syn are “gain of function”, and favoring a “loss of MAMs function” hypothesis (Guardia-Laguarta et al., 2014, 2015). Additional data support defective MERCs contribution to the etiology of PD: mutants for parkin (PARK2), DJ-1 (PARK7), and PINK1 (PARK6), all causing recessive early-onset PD cases, can impact on ER-mitochondria tethering, mitochondrial quality control, and Ca^2+^ transfer between the two organelles (Li et al., 2005; Narendra et al., 2008; Davison et al., 2009; Ziviani et al., 2010; Calì et al., 2013).

Mutated MERCs proteins are not the only reason for the development of MERCs linked diseases or symptoms: being “raft”-like domains, changes in lipid homeostasis could exert deleterious effects on their structure/composition. One of such example could be atherosclerosis. It has been shown that an ER overload of cholesterol in murine macrophages causes prolonged ER stress and UPR activation culminating in apoptosis, substantially contributing to the progression of this disease (Tabas, 2002; Feng et al., 2003). An intriguing question is whether MERCs are modulated in arterial wall cell populations at different stages of atherosclerosis progression, and what their pathogenic impact may be. Notably, MERCs could also take part to the inflammatory response involved in such pathologies, as they have also been involved in the activation of the inflammasome complex and ILβ production, although this needs to be more deeply investigated (Zhou et al., 2011; Lerner et al., 2012; Marchi et al., 2014).

Altogether these findings suggest that defective MERCs localization is likely not only to constitute discrete, primary elements of pathogenesis, but also to be a source of epistatic effects underlying the impact of additive risk factors.

## Conclusions

In the last years the interest on MERCs biology has exponentially grown, due to the evidence that at these interfaces many biological processes integrate and that MERCs defects underlie several pathological conditions. Many proteins have been retrieved in the biochemical counterparts of MERCs (that are, MAM fractions, see above) but so far the mechanisms responsible for targeting at MERCs have yet not been fully elucidated. Interestingly, post-translational modifications such as palmitoylation, miristoylation, and oxidation seem to gain the upper hand over a more canonical targeting signal. Another standing question pertains as to how special conditions, such as ER stress, specifically contribute to determine MERCs composition and hence functional state. It is likely that several independent features of an ER-stressed cell take part to such remodeling. Acute ER stress is frequently associated with alteration (mostly *attenuation*) of signaling pathways which are considered to stabilize MERCs, namely AKT-mTOR signaling (Betz et al., 2013), or with increased ER Ca^2+^ levels, that could in turn enhance (at least in early stress phases) mitochondria-ER proximity as an adaptive response for the maintenance of intracellular Ca^2+^ homeostasis (Bravo et al., 2011). Chronic ER stress as well can induce MERCs remodeling: for example, lipid imbalance associated with obesity might promote connectivity between the two organelles, in an attempt to restore equilibrium among different lipid species and to exert a tighter control on Ca^2+^ homeostasis, which is significantly perturbed in such dyslipidemic states (Fu et al., 2011; Arruda et al., 2014).

Overall, despite a small number of elegant studies on MERCs targeting mechanisms and protein relocation at MAM fractions have been published (such as for example, Myhill et al., 2008; Lynes et al., 2012), this aspect in the field of MERCs biology appears to be just at its infancy. Exciting findings lie ahead, and their discovery will certainly represent another step forward into the complexity of cellular signal transduction, as well as in the understanding of pathological processes.

## Author contributions

MG, MS-Á, and MD: conceived, designed, and wrote the manuscript; NI, MB, and VC: drafted and revised the article; MS-Á and VC prepared figures.

### Conflict of interest statement

The authors declare that the research was conducted in the absence of any commercial or financial relationships that could be construed as a potential conflict of interest. The reviewer IB and handling Editor declared their shared affiliation.

## Abbreviations

AD: Alzheimer's disease
ASC: apoptosis-associated speck-like protein containing a CARD
ATF4/6: activating transcription factor 4/6
BIM: Bcl2-interacting mediator of cell death
CHOP: C/EBP-homologous protein
CNX: calnexin
DGAT2: diacylglycerol O-acyltransferase 2
eIF2: eukaryotic translation initiation factor 2
ER: endoplasmic reticulum
ERAD: ER-associated degradation
ERMES: endoplasmic reticulum (ER)-mitochondria encounter structure
FACL4: fatty acid-CoA ligase 4
GRP75: 75 kDa glucose-regulated protein
IMM: inner mitochondrial membrane
IP3R: inositol triphosphate receptor
IRE1: inositol requiring enzyme 1
MAM: mitochondria-associated membrane
MERC: mitochondria-ER contact site
MFN1/2: Mitofusin 1/2
MPAN: mitochondrial membrane protein associated neurodegeneration
mTOR: mammalian target of rapamycin
OMM: outer mitochondrial membrane
PACS2: phosphofurin acidic cluster sorting protein 2
PC: phosphatidylcholine
PD: Parkinson's disease
PE: phosphatidylethanolamine
PERK: protein kinase R-like endoplasmic reticulum kinase
PI(3,5)P2: phosphatidylinositol 3,5-bisphosphate
PI3K: phosphoinositide 3-kinase
PrP: prion protein
PS: phosphatidylserine
PSD: phosphatidylserine decarboxylase
PSS1/2: phosphatidylserine synthase 1/2
PTEN: phosphatase and tensin homolog
PTM: post-translational modifications
PTP: permeability transition pore
PTPIP51: protein tyrosine phosphatase interacting protein 51
PUMA: p53 up-regulated modulator of apoptosis
REEP1: receptor expression-enhancing protein 1
ROS: reactive oxygen species
RRBP1: ribosome-binding protein 1
S1/2P: site 1/2 protease
SMP: synaptotagmin-like mitochondrial-lipid binding protein
STBD1: starch binding domain-containing protein 1
SYNJ2BP: synaptojanin-2-binding protein
TMX: thioredoxin-related transmembrane protein
TXNIP: thioredoxin-interacting protein
UPR: unfolded protein response
VAPB: vesicle-associated membrane protein associated protein B
VDAC: voltage-gated anion channel
XBP1: X-box binding protein 1.
